# Codon usage and modular interactions between messenger RNA coding regions and small RNAs in *Escherichia coli*

**DOI:** 10.1186/s12864-018-5038-6

**Published:** 2018-09-06

**Authors:** Mario Tello, Felipe Avalos, Omar Orellana

**Affiliations:** 1 Centro de Biotecnología Acuícola, Departamento de Biología, Universidad de Santiago de Chile, Alameda 3363, 9170022 Estación Central, Chile; 2 Programa de Biología Celular y Molecular, Instituto de Ciencias Biomédicas, Facultad de Medicina Universidad de Chile. Independencia 1027, 8380453 Santiago, Chile

**Keywords:** sRNA, Codon usage, Modular structure, Interacting coding regions

## Abstract

**Background:**

Small RNAs (sRNAs) are key regulators of gene expression in bacteria. In addition to modulating translation initiation, sRNAs can interact with mRNA coding regions to regulate mRNA stability and translation efficiency, enhancing or impeding progression of the ribosome along the mRNA. Since most amino acids are decoded by more than one codon (synonymous) we asked as to whether there is a codon bias in the interaction of sRNAs with coding regions of mRNAs. Therefore, we explored whether there are differences in codon usage or tRNA availability according to whether an mRNA is regulated by sRNAs or not. We also explored these parameters in the coding interaction regions in mRNAs. We focused our analysis on sRNAs that regulate multiple mRNAs.

**Results:**

We found differences in codon adaptation index and tRNA adaptation index between sRNA-regulated and non-sRNA-regulated mRNAs. Interestingly, the sRNA-mRNA interacting regions tended to be enriched in unpreferred codons decoded by scarce tRNAs. We also found that sRNAs with multiple targets often contained modular segments capable of recognizing conserved motifs among these mRNAs.

**Conclusions:**

Our results show that sRNAs in *E. coli* tend to recognize mRNA coding regions in which the ribosome is predicted to advance at low speeds. Identified motifs in interacting regions are conserved among mRNAs that are recognized by the same sRNA.

**Electronic supplementary material:**

The online version of this article (10.1186/s12864-018-5038-6) contains supplementary material, which is available to authorized users.

## Background

Small non-coding RNAs (sRNAs) are crucial regulators of gene expression in bacteria. These 50- to 500-nucleotide-long RNAs are encoded in the genomes of all known bacteria and play major roles in many cellular processes, particularly in response to stress conditions [1, 2]. The major role of sRNAs is regulation of translation initiation, achieved by interfering with interactions between ribosomes and their binding sites at the 5’untranslated region (UTR) of the target mRNA and/or the very first codons of the open reading frame. sRNAs can also modulate mRNA stability and processing. These functions are exerted through imperfect base pairing with the target mRNA, initiated by the interaction of a few seed nucleotides followed by other base pairings. The RNA chaperone Hfq plays a major role in facilitating and stabilizing many of these interactions between these two RNA partners [3]. This Sm-like, homohexameric protein interacts with RNAs primarily through recognition of U-rich sequences and RNA motifs in the sRNA and target mRNA, respectively. Hfq-assisted recruitment of RNAse E to the base-pairing region results in degradation of the mRNA. Conversely, other interactions between an sRNA and its target block the action of RNAses, thereby stabilizing the mRNA.

The role of sRNAs in regulatory networks, particularly in modulation of transcription, has gained increasing recognition in recent years. How transcription and translation of the genetic information are coordinated with the participation of sRNAs in response to cellular cues is a topic of intense research [4]. It has been reported that a single mRNA can be the target of multiple sRNAs with different regulatory modes. This discovery shed critical light on the conformation of regulatory networks, particularly given that some mRNA targets may serve as regulatory molecules and transcription factors. The finding that one mRNA might be the target of several sRNAs also suggests that cross-talk among various cues may influence the fate of an mRNA and therefore translation of the encoded protein. Understanding the global effects of each sRNA might contribute to elucidating the general mechanisms of cellular response to changing environmental conditions.

Predictive and experimental approaches have been used to identify the mRNA targets of sRNAs. A number of computational tools to detect the complementary regions of sRNAs and mRNAs have been developed, including tools that make use of energy as well as phylogenetic criteria to discriminate between true and false predictions [5–8]. Experimental global approaches have also been developed to identify Hfq-mediated sRNA-mRNA pairings [9]. Most of the experimentally-reported target sites for sRNAs are located in the 5’UTR of the mRNAs, which is consistent with the well-established role of sRNAs in regulating translation initiation. However, an increasing number of interactions between sRNAs and mRNA coding regions have been reported recently as well [9, 10], including experimentally-confirmed examples. Most reported cases involve complexes consisting of sRNA-mRNA duplexes and Hfq, indicating that this chaperone plays a major role in modulating interactions involving mRNA coding regions.

As interactions between sRNAs and target mRNAs require base complementarity, interactions with mRNA coding regions may be influenced not only by sRNA-mRNA sequence complementarity but also by the synonymous codons encoding the amino acids of the protein.

Therefore, codon usage bias may add another level of restriction to this interaction. Most amino acids are encoded by more than one codon (often 2, 4, or 6 codons); however, these codons are not evenly distributed throughout the genome, the chromosome, or even the gene. This non-random distribution represents the so-called codon usage bias [11]. The basis of this bias at different structural levels is not yet understood. At the gene level, codon bias may be implicated in translation efficiency and therefore protein folding [12]. Therefore, both the amino acids encoded and the codon bias in the interaction region of the mRNA may influence pairings with sRNAs.

To assess this hypothesis, we developed a global prediction of mRNA-sRNAs pairings occurring in mRNA coding regions in *E. coli*. Codon usage and predicted availability of tRNA were determined. Special emphasis was placed on sRNAs that interact with multiple mRNAs, as conservation of interaction regions is expected, constraining the locations and characteristics of interactions potentially involved in network conformation.

## Methods

### sRNA and mRNA target sequences from *E. coli* K12 MG1655

The identities and sequences of the sRNAs described for *E. coli* K12 MG1655 were downloaded from the Bacterial Small Regulatory RNA Database (BSRD database) [13]. The identities of the target mRNAs reported for these sRNAs in *E. coli* K12 MG1655 were also obtained from the BSRD database. The sequences of the coding regions, 5’UTR (untranslated region), and 3’UTR were downloaded from EcoGene 3.0 (https://www.re3data.org/repository/r3d100010546) [14]. The UTRs for each gene were defined as the 50 nucleotides upstream or downstream from the start or stop codon, respectively. Targets of sRNAs not assigned in the BSRD database were predicted using RNApredator [6].

### Identification and prediction of sRNA-mRNA interacting regions

The sequences of empirically-determined sRNA-mRNA interaction regions were obtained from sRNATarBase 3.0 [15]. Uncharacterized interaction regions were predicted using IntaRNA software [16]. For all interactions, the **Δ**G of binding was predicted using IntaRNA software.

### Determination of CAI and tAI

The codon adaptation index (CAI) was calculated for each coding sequence using JEmboss [17]. To calculate the CAI for the region within the mRNA coding sequence that interacts with the sRNA, the region was identified using IntaRNA, and the minimal set of extra nucleotides required to conserve the original open reading frame was extracted. These sequences were then analyzed using JEmboss. To calculate adaption to the tRNA pool, we calculated the tRNA adaptation index (tAI) using a Python script [18]. To calculate tAI for the regions within the mRNA coding sequence that interact with sRNA, we extracted the sequences as described above and analyzed the sequences using a tAI calculation script.

### Analyses of RIL-seq data

Information about the region of interaction and genes involved in the Hfq complex was obtained from the Additional file 1 from Melamed et al. report [9]. Coordinates and sense from interaction regions reported in this file were used to extract the sequences from *E. coli* MG1655 genome with a script written in Python language. Then to analyze only the region that encodes for complete codons, the sequences were analyzed using BLASTX against the protein sequences encoded in *E. coli* MG1655 genome to obtain the coordinates of coding sequences. This information was used to re-edit the sequences taking into account that all sequences must have a size that is a multiple of 3. CAI and tAI values were calculated according to the methods described above.

### Interaction density maps for sRNAs with multiple targets

For each sRNA that recognized more than four mRNA targets, we developed a density map of interactions with mRNA targets along the sRNA sequence. In brief, each sRNA nucleotide was assigned a value of 1 when it interacted with an mRNA and a value of 0 when it did not. The sums of all positive interactions for each sRNA position were used to develop interaction profiles, in which a higher total score indicated that the nucleotide interacted with a greater number of mRNAs, and a total score of zero meant that the nucleotide did not interact with any mRNA.

### Identification of conserved mRNA regions that interact with the same sRNA

Conserved regions among the different mRNAs that recognized a common sRNA were identified using the interaction density maps. In brief, the density maps indicated which mRNAs primarily interacted with a single sRNA subzone or with two or more subzones. The mRNA sequences that interacted with each subzone were determined using IntaRna software and extracted manually. The groups of sequences that recognized a single sRNA zone or multiple subzones were analyzed using MEME Package to identify conserved motifs (http://meme-suite.org/tools/meme) [19]. We used a *p*-value of < 0.05 as cutoff value for a statistically-significant motif.

### Network identification

The sRNA-mRNA networks present in *E. coli* K12 MG1655 were identified and visualized using Cytoscape software [20]. sRNAs and mRNAs were represented as nodes. Edges connecting sRNA and mRNA nodes represented interactions between sRNAs and mRNAs. For networks involving an sRNA with multiple target mRNAs, edges connecting the sRNA zone and subzone to other subzones or the target mRNA also represented interactions.

## Results

### Identification of interacting regions in mRNAs regulated by sRNAs

**Fig. 1:**
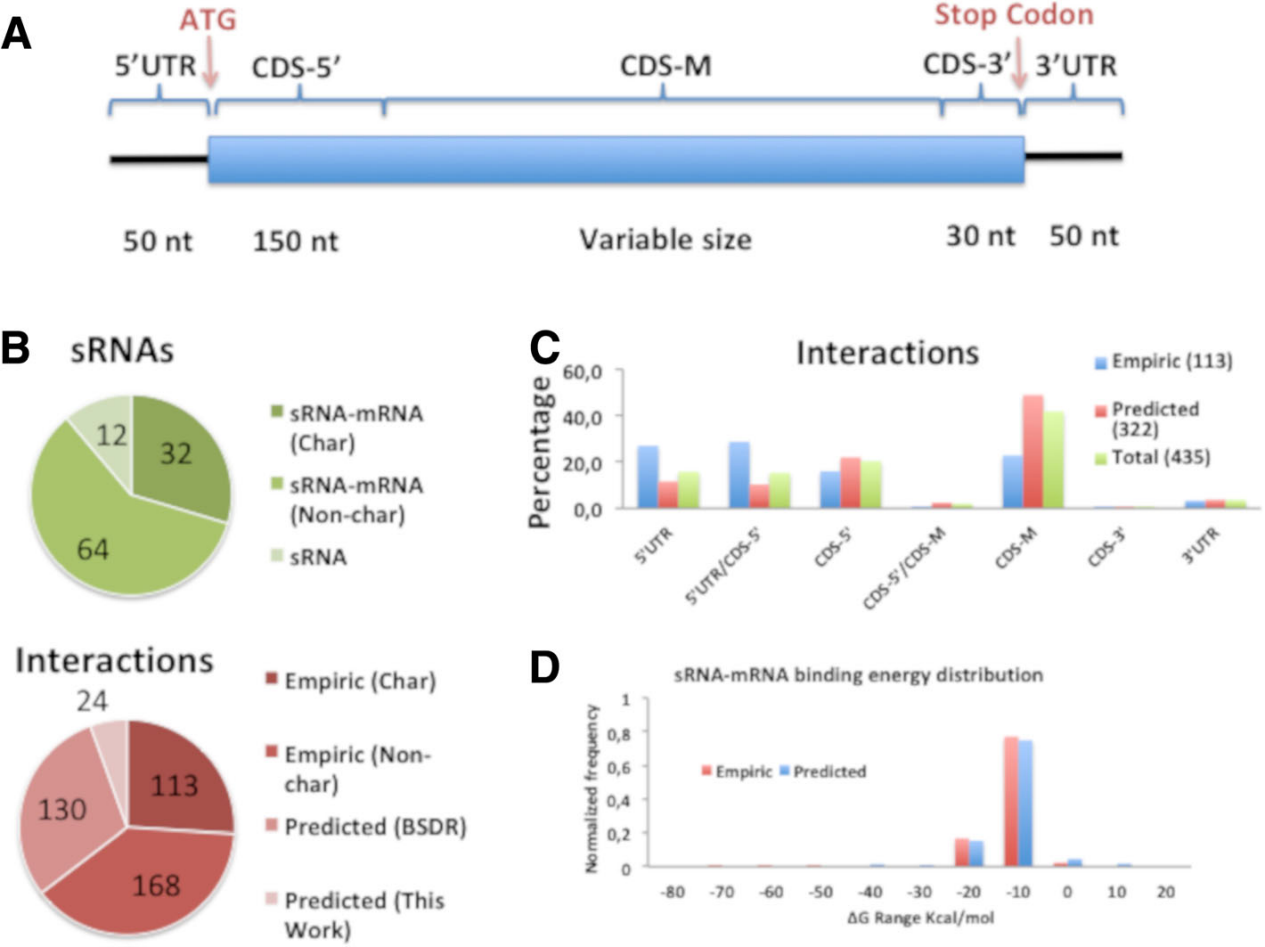
Status of sRNA-mRNA interaction data in *E. coli* K12 MG1655. **a** Schematic representation of the arbitrary subdivision of mRNAs used in this work. 5’-UTR, coding DNA sequence (CDS-5’, middle (CDS-M), CDS-3’), 3’-UTR). **b** Upper panel: sRNAs archived in BSRB were classified according to the status of knowledge regarding their interactions with target mRNAs: characterized interaction (Char), uncharacterized interaction (Non-char), or unknown (sRNA) (Fig. 1B). Lower panel: interactions found in BSRD were also classified as empirical or predicted according to the method used for their determination. **c** Comparison of the distributions of predicted (this work) and characterized (BSDR) interactions of the various segments of the mRNAs. **d** Distributions of empirical and predicted binding energies of sRNA-mRNA interactions. Nt indicates for nucleotides

Current information about sRNA and their targets are deposited in databases such as BSRD (http://www.bac-srna.org/BSRD/index.jsp) [13]. According to BSRD, a total of 108 sRNAs are reported in *E. coli* K-12 MG1655, and 96 of which are known to interact with 304 mRNAs, resulting in 412 sRNA-mRNA interactions. Our analysis of this database identified three types of sRNA: a) sRNAs with no identified targets, b) sRNAs with identified targets, but no characterized sRNA-mRNA interactions, and c) sRNAs with identified target(s) and characterized sRNA-mRNA interaction(s). Approximately 26.0% of the interactions (113) involved complexes in which the mRNA-sRNA interaction region had been localized, while 74.0% (191) involved interactions in which only the existence of sRNA-mRNA pair was known. These interactions were classified as “characterized” or “uncharacterized,” respectively. A total of 64 sRNAs had uncharacterized and 32 had characterized interactions. As only 26% of the interactions were characterized at the level of the sRNA-mRNA, we chose to characterize the remaining interactions. We predicted the binding regions for all uncharacterized interactions using IntaRNA software [16] (Additional file 2). Interactions were then classified according to the location of the binding region in the target mRNA. Empirically-documented interactions were most commonly found in the 5’UTR or adjacent to the 5’ end of the coding DNA sequence (CDS). Although some predicted interactions also occurred in these regions, most were found to occur in the middle of the coding regions (Fig. 1). This observation is consistent with the RNA-seq characterization of Hfq-dependent sRNA-mRNA interactions described by Melamed et al. [9, 21], in which approximately 60% of all described interactions occur in the middle of the coding regions.

To verify that the predicted interactions represented plausible models, we compared the predicted and empirically-determined **Δ**G of interaction. The results showed that the binding energy of both types of interactions were within the same range (Fig. 1d).

### Modular interactions of sRNAs with multiple mRNA targets

**Fig. 2:**
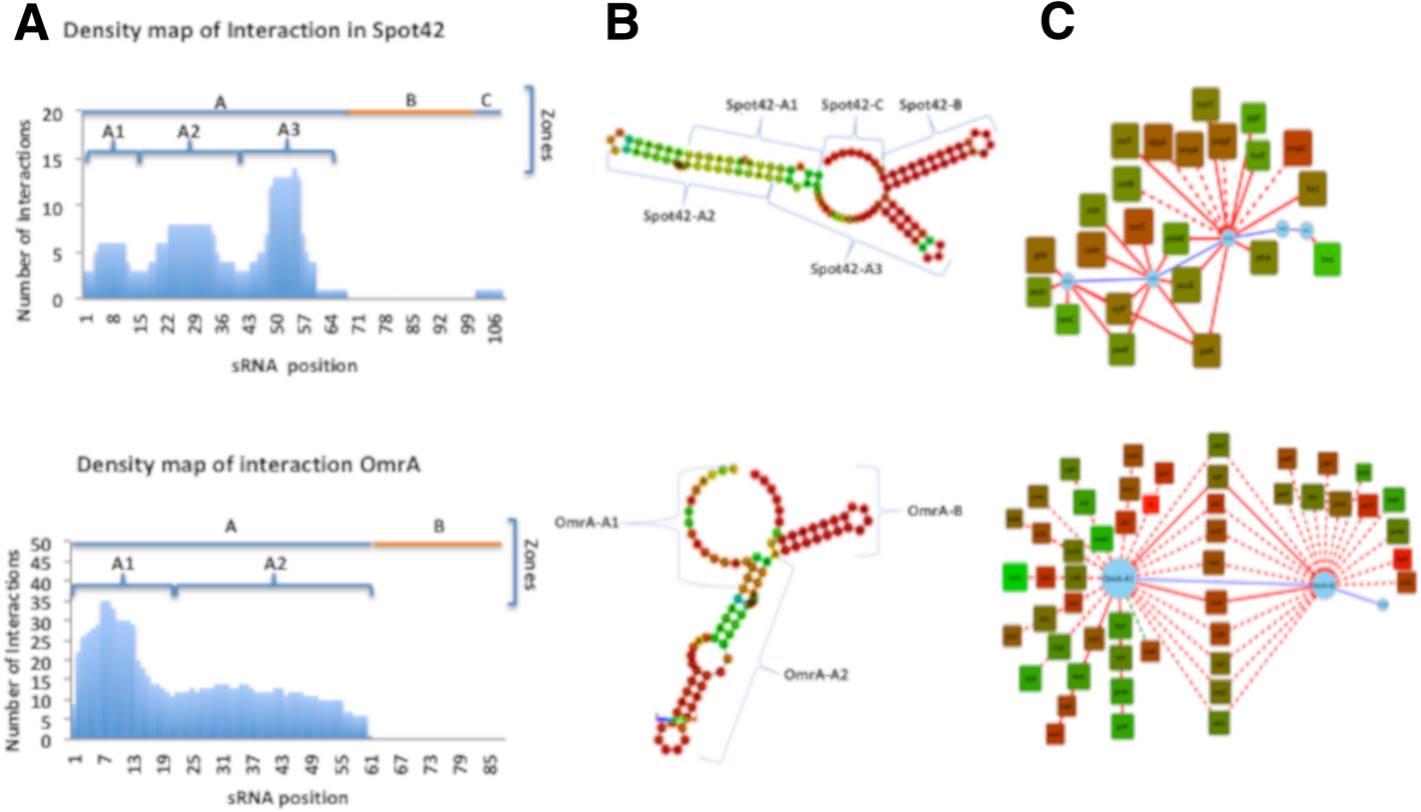
Modular structure of sRNAs with multiple targets. **a** The numbers of interactions for Spot42 (top panel) and OmrA (bottom panel) were plotted as a function of the position of each nucleotide in the primary sequence of the sRNA, allowing for construction of a density map that was then used to identify the zones (top line) and subzones (brackets) that interact with mRNAs. **b** Interaction zones and subzones were located in the predicted secondary structures of the sRNAs. The probability of forming a base pair is indicated using a color scale ranging from 0 (green) to 1 (red). **c** A network of the interactions between the mRNA and the sRNA interaction zones was then developed (Panel **c**). Blue circles connected by blue lines represent the sRNA structure. Boxes represent the mRNA, and edges represent interactions with the corresponding sRNA zone. Repression (red lines), empirical (solid line), and predicted (dotted line) interactions are indicated. The CAI values for each target are indicated using a color scale ranging from green (low CAI values) to red (high CAI values)

To understand how sRNAs interact with multiple targets, we identified sRNAs with more than four targets and mapped the nucleotides that interact with the targets for each case (Fig. 2). This analysis revealed that a total of 16 sRNAs interacted with at least four mRNAs (Table 1). The density of interactions (measured as the number of interactions per nucleotide) along the sRNA sequence allowed for identification of zones and subzones that interact with defined groups of mRNAs. This analysis also showed that some mRNAs interacted with more than one subzone (Fig. 2c). Most sRNAs contained at least two zones, one or more interacting with mRNAs and another that had a high probability of forming a stem-loop structure (Fig. 2b). Only RyhB was found to interact with mRNAs along its entire sequence. RygD, RprA, SgrS, DsrA, OxyS, and GcvB showed more than one zone with a high probability of forming a stem-loop secondary structure that would not interact with any mRNA (Table 1, Additional file 1). Analyses focused on interactions within the coding regions produced similar profiles (Additional files 3 and 4). To determine whether the different zones and subzones of each sRNA have a preferred interaction region, we localized the interactions within the target mRNA sequences. Our results showed that each sRNA was capable of interacting with different regions on the target mRNAs (5’UTR, 5’UTR/ -CDS, CDS-5, CDS-M, 3’CDS CDS-3, and 3’UTR); however, sRNA zones and subzones located at the ends of the sequence (5’ or 3’) tended to recognize particular regions of the mRNA. The sRNA OmrA provides an example of the first pattern of interaction. About 89% of sRNA-mRNA interactions (25/28) involving subzone OmrA-A1 occurred in the CDS-M, while 60% (6/10) of interactions involving subzone OmrA-A2 occurred in the 5’UTR of the target mRNA. The sRNAs RyhB, OmrB, GcvB, and CyaR showed a similar pattern. OxyS exemplified the opposite pattern; 67% of interactions involving OxyS-A occurred in the 5’UTR, while 70% of interactions involving OxyS-C2 occurred in the CDS of the target mRNA. Spot42, FnrS, DsrA, MicF, and RygD shared this pattern (Additional file 5). These results suggest that sRNAs with multiple targets have a modular structure, in which each subzone primarily recognizes the same type of region in their target mRNAs.

**Table 1:** Distribution of interactions of sRNAs that recognize multiple mRNA targets

sRNA	Total number of targets	Zones (number of interactions)
OmrA	50	A1 (39), A2 (21), B (0)
RyhB	43	A1 (16), A2 (33), A3 (8)
OmrB	26	A1(19), A2(18), B (0)
GcvB	26	A (19), B (0), C (7), D (0)
Spot42	23	A1 (6), A2 (8), A3 (14), B (0), C (1)
OxyS	18	A (8), B (0), C1 (3), C2 (9), D (0)
MicA	14	A (14), B (0)
RybB	11	A (11), B (0)
FnrS	10	A1 (2), A2 (8), A3 (3), B (0)
DsrA	10	A (0), B1 (6), B2 (8), B3 (1), C (0)
SgrS	8	A (0), B (8), C (0)
MicC	8	A (2), B (0), C (6), D (0)
MicF	7	A1 (5), A2 (2), C (0)
RprA	6	A (0), B (6), C (0)
CyaR	4	A1 (2), A2 (3), B (0)
RygD	4	A (0), B (1), C (0), D (1), E (0), F (1), G (0), H (1), I (0)

The table shows the list of sRNAs with multiple targets (> 4), the total number of interactions, the zones (or subzones) identified with the density map analysis, and the number of interactions by zone (or subzone).

### sRNAs that interact with multiple targets recognize regions that are conserved among sRNA-regulated mRNAs

**Fig. 3:**
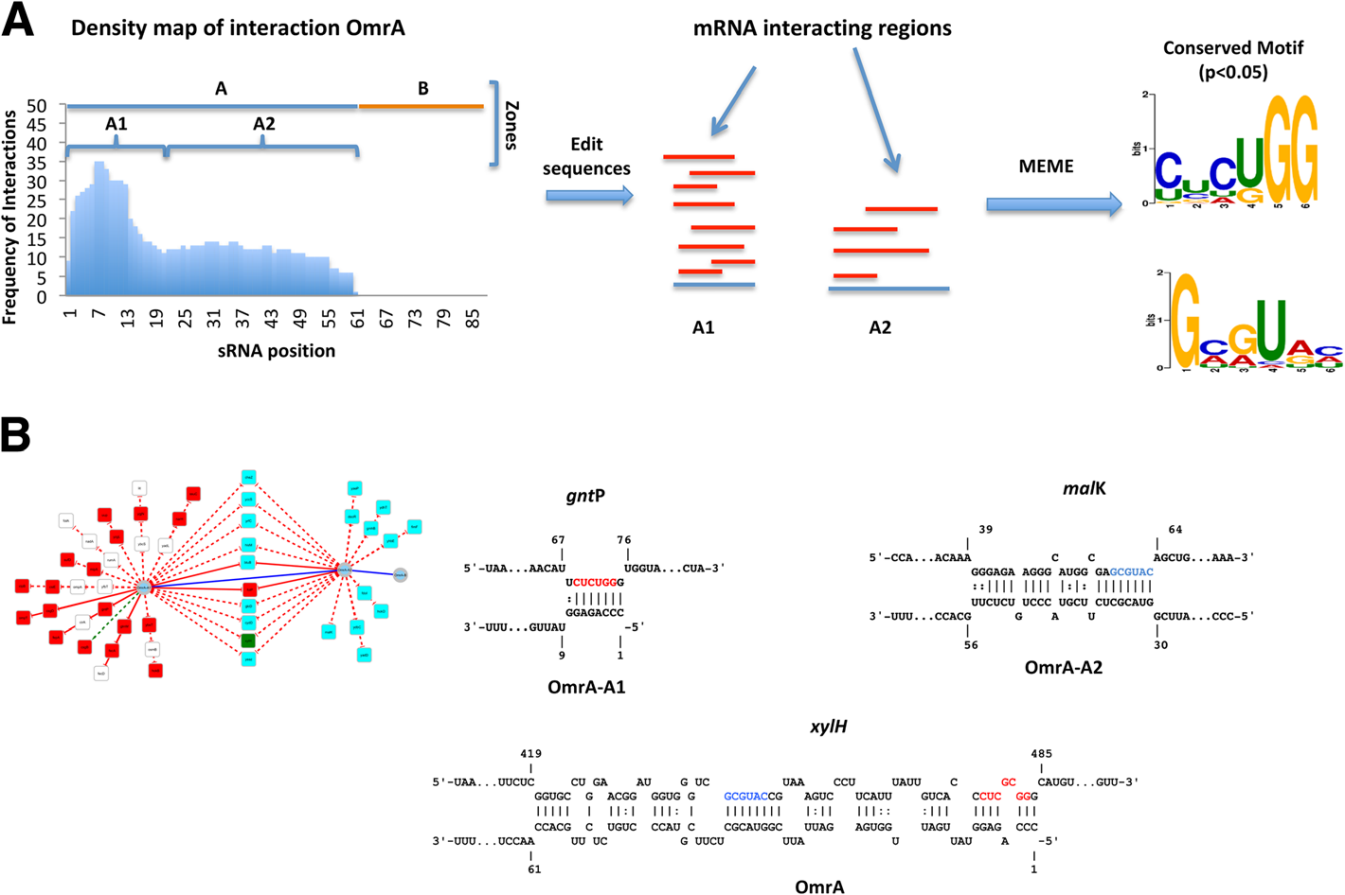
Strategy for identifying conserved regions in mRNAs recognized by a common sRNA**.** As an example, the interactions between OmrA and mRNAs containing one conserved motif (*gnt*P and *mal*K) or two conserved motifs (*xyl*H) are shown. **a** To identify conserved mRNA regions that recognize a common sRNA, we first constructed an interaction density map. The mRNA sequence segments recognized by zones or subzones of a particular sRNA were then determined based on these density maps. These short sequences were aligned and analyzed using MEME to identify conserved motifs. Significantly conserved motifs are represented by a sequence logo. **b** Complimentary interactions between sRNAs and mRNAs were predicted, and the conserved motifs were mapped in the mRNA. A network approach was used to graphically represent the presence of conserved motifs in the mRNAs that interacted with the sRNA zone and subzones (nodes and edges in blue). mRNAs with conserved motifs complementary to the A1 subzone, the A2 subzone, or both are shown as red, cyan, and green nodes, respectively

The results presented in the previous section suggest that sRNAs with multiple targets have a modular structure with zones and subzones that are specialized for interaction with particular mRNA regions, including the coding sequences. To determine whether the subzones identified recognized conserved regions of target mRNAs, we analyzed the sequences of the mRNAs that interacted with each subzone (Fig. 3). We found conserved sequences (statistically significant with *p* value < 0.05) in target mRNAs that were recognized by each of the following sRNAs: OmrA, RyhB, GcvB, OxyS, and Spot42. (Table 2). We evaluated whether these conserved sequence motifs were complementary to the cognate sRNA subzones. Our analysis shows that these motifs were complementary to the sRNA subzones, indicating that interaction with sRNA is likely the major role of these regions. This result supports the idea that sRNAs with multiple targets interact with target mRNAs through modules conserved in sequences, which allow for sRNA-mRNA interactions based on complementarity. These motifs were located mainly in coding regions (5’UTR (17%), 5’UTR-CDS (21.3%), CDS-5’ (21.3%), CDS-M (36.2%), CDS.3’ (1%), and 3’UTR (3.2%)), suggesting that constraints induced by the coding sequences influence sRNA-mRNA interactions through conserved motifs.

**Table 2:** Conserved mRNA motifs that recognize modular sRNA sequences

sRNA^(a)^	*p*-value^(b)^	Motif size^(c)^	Targets^(d)^	Motif^(e)^	Number of sequences^(f)^
OmrA-A1	9.6 × 10^−10^	6	21 (*gnt*P, *ygj*N, *hok*B, *ssu*C, *csg*D, *ybe*T, *mip*A, *clp*B, *yzg*L, *omp*T, *nar*H, *fec*A, *xyl*H, *fep*A, fecD, *uup*, *csg*B, *csi*E, *fol*P, *suf*D, *glm*M)		21/39
OmrA-A2	7.9 × 10^−3^	6	20 (*mal*K, *gmh*B, *xyl*H, *yrf*C, *deo*R, *btu*B, *ycc*S, *cyd*D, *glc*D, *hok*D, *yhb*E, *yae*P, *che*Z, *yea*Z, *yad*D, *his*M, *ydh*T, *ydb*C, *fdo*I, *fim*F)		20/20
RyhB-A2	8.9 × 10^−13^	8	19 (*suc*D, *pro*A, *ygi*Q, *yag*J, *yeg*K, *yia*M, *bfr*, *yda*N, *suc*B, *sug*E, *yad*S, *met*H, *ygi*T, *mdh*, *met*I, *cit*G, *yag*T, *fum*A, *shi*A)		19/33
RyhB-A3	1.4 × 10^−3^	8	5 (*omp*C, *gal*K, *met*I, *sod*B, *isc*S)		5/8
GcvB-A	7.3 × 10^−6^	10	17 (*opp*A, *ybd*H, *ilv*C, *liv*K, *thr*L, *omp*A, *ici*A, *omp*F, *dpp*A, *omp*C, *sod*B, *ndK*, *liv*J, *pts*G, *yae*C, *glt*I, *sst*T)		17/19
Spot42-A1	5.1 × 10^−5^	8	6 (*glt*A, *ato*D, *nan*C, *paa*K, *gal*K, *xyl*F)		6/6
Spot42-A3	1.7 × 10^−11^	8	10 (*fuc*P, *omp*C, *omp*A, *glp*F, *fuc*I, *omp*F, *puu*E, *asc*F, *dpp*A, *gal*K)		10/14
OxyS-A	1.4 × 10^−2^	7	6 (*yccE, gfcB, yheN, yobF, yeaK, moaD)*		6/6

The table shows the motifs identified in the region of interaction mRNA-sRNA present in target mRNAs. The table indicates the sRNA zone of interaction^(a)^, *p*-values of identified motifs^(b)^, size of motifs^(c)^, number and identity of target mRNAs containing the identified motifs^(d)^, logo representation of motifs^(e)^ and the fraction of target mRNAs containing the identified motifs^(f)^

Our analysis found 5 sRNAs (OmrA, RhyB, GcvB, Spot42, and OxyS) that interacted with significantly conserved mRNA motifs. Melamed et al. [9] previously described 16 sRNAs with target motifs, but their list did not include OmrA, Spot42, or OxyS. Despite the different approaches, we identified the same target motifs reported by Melamed et al. for GcvB and RyhB. Additionally, our study included an analysis of interactions between specific mRNA regions and sRNA subzones. In fact, splitting the analysis into sRNA subzones increased the number of motifs identified (Fig. 3, Table 2).

This analysis also showed that mRNAs that interact with two or more sRNA subzones generally do so via a single motif. In fact, the mRNAs encoding for XylH, GalK, and MetI were the only identified examples of mRNAs containing two motifs, each recognizing a specific sRNA subzone.

For example, the OmrA-A1 subzone interacted with 39 mRNAs, and 21/39 contained the 6 bases motif CUCUGG. On the other hand, the OmrA2 subzone interacted with 20 mRNAs, all of which contained the 6 bases consensus motif GCGUAC. Of the 10 mRNAs that interacted with these two subzones, *xyl*H was the only mRNA with interactions involving both consensus motifs.

RyhB was the sRNA with the second-largest number of interactions. We identified a motif in approximately 50% of the mRNAs regulated by this sRNA. This consensus sequence was located in the RyhB-A2 subzone, where the MEME package also localized a motif in 19/33 mRNAs that interacted with this region. RyhB also shows a conserved motif in 5/8 mRNAs that interacted with the RyhB-A3 subzone. *met*I was the only mRNA that interacted with RyhB through the RyhB-A2 and RyhB-A 3 motifs.

For Spot42, we identified a motif in 100% of the mRNAs that interacted with the Spot42-A1 subzone. A consensus motif was identified in 71% of the mRNAs that interacted with Spot42-A3. GalK was the only gene that showed a conserved motif for interactions with the Spot42-A2 and Spot42-A3 subzones (Table 2).

Our analysis also showed that there are two classes of sRNA subzones, a “primary” subzone that contains the “seed” sequence for interaction with the conserved mRNA motif and a “secondary” subzone that stabilizes the interaction (data not shown).

### Codon usage of sRNA-regulated mRNAs and adaptation to the predicted tRNA pool

**Fig. 4:**
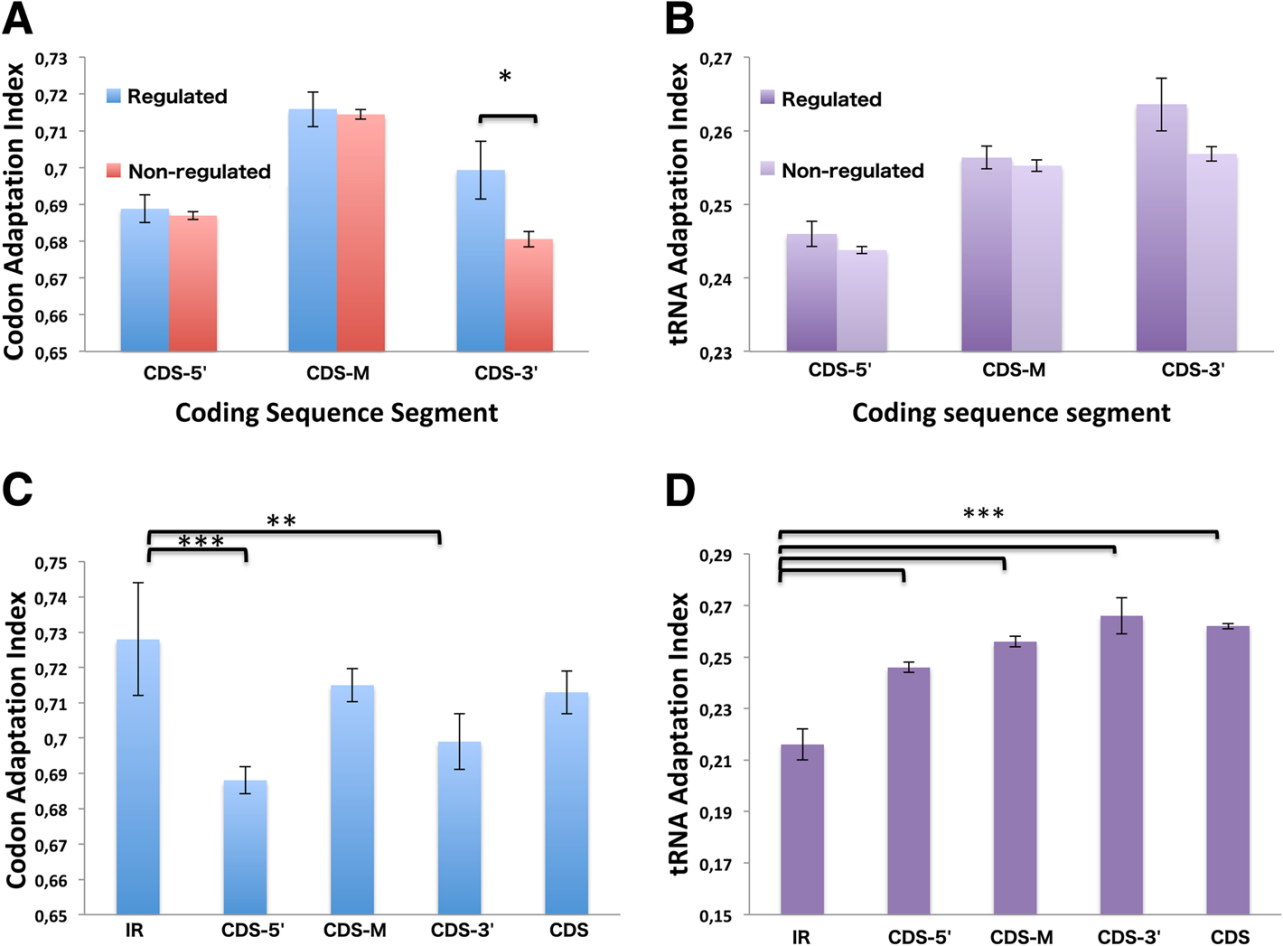
Codon preferences in sRNA-mRNA interactions. The mRNAs regulated by sRNAs were characterized according to codon usage (Codon Adaptation Index) (**a**) and availability of tRNAs to decode the mRNA (tRNA Adaptation Index) (**b**). sRNA-mRNA interaction regions (IR) located within the coding sequence were also characterized according to codon usage (**c**) and tRNA availability (**d**). Statistical significance was assessed using the Mann-Whitney test, *0.05 > *p* > 0.01, **0.01 > *p* > 0.001, ***p < 0.001

Once the target regions in the predicted sRNA-regulated mRNAs were identified, we asked whether these mRNAs might show a different codon composition pattern than non-sRNA-regulated mRNAs. To perform this analysis, we compared the codon adaptation index (CAI) of both groups of mRNAs. No significant differences between the groups were found for CAI values calculated using the complete coding sequence (data not shown). However, splitting the coding sequence into 3 regions (CDS-5’, CDS-M, and CDS-3’, shown in Fig. 1a), a small, statistically significative difference between the groups (*p* = 0.0217) was identified in the C-terminal coding region, where the sRNA-regulated mRNAs showed slightly higher CAI values (Fig. 4a). A similar result was observed for adaptation to the tRNA pool (tAI) (Fig. 4b). Our results showed that sRNA-regulated mRNAs did not show any special codon preference pattern as compared to mRNAs not regulated by sRNAs. The absence of a significant difference could be the consequence of the reduced number of interactions analyzed. Currently, the data presented by Melamed et al. [9] represent the most extensive empirical information reported for sRNA-mRNA interactions in *E. coli.* Based on this information concerning the sRNA-mRNA interactions mediated by the RNA chaperone Hfq, we found that genes that are part of Hfq complexes tend to have slightly higher values of CAI (0.7186 ± 0.0015, *n* = 1155) compared to genes that do not form complex (0.7034 ± 0.001054, *n* = 3164) with a *p* value < 0.0001 using Mann-Whitney test. Similar results were obtained for tAI values of genes forming complexes with Hfq (0.2602 ± 0.001) compared to those that do not form complex (0.2536 ± 0.0001).

As the free energy of the sRNA-mRNA interaction depends on the length of the sequence and GC content of the interacting segments, it is possible that certain codon biases could be involved in the determination of interacting regions (IR). The CAI values for sRNA-mRNA interacting regions were compared to CAI values for the entire CDS (or other segments, as shown in Fig. 1a) in sRNA-regulated mRNAs. We found that the interacting regions tended to use the most-abundant codons (higher CAI values) (Fig. 4c). Although small differences were observed between the interacting regions and the entire coding region, statistically-significant differences were observed only for the first 150 (CDS-5’) or the last 30 nucleotides (CDS-3’) of the coding region. This result could reflect the previously-described translational ramp, which prevents the ribosome stalling [22]. When adaptation to tRNA availability was predicted, (Fig. 4d), the results showed that the interacting regions had lower tAI values vs. the coding sequence as a whole and vs. other segments (according to Fig. 1a). These results suggest that sRNAs tend to interact with mRNA coding regions with limited availability of tRNAs to decode them. When these analyses were performed using the information reported by Melamed et al. [9], we found similar low tAI values for the interacting regions (0.2062 ± 0.001), however CAI values were lower (0.6160 ± 0.001) (Additional files 6 and 7). Since these values were obtained using a much larger sample (*n* = 3580), they strongly support the information provided by the BSRD platform. Lower CAI values are more consistent with low tAI values found for interacting regions using either BRSD or Melamed et al. data, reinforcing the idea that these regions have a codon bias deviated to unpreferred codons.

Altogether, these findings suggest that ribosomes may tend to pause at the sRNA-mRNA interacting regions. Whether the ribosome protects the mRNA against degradation by RNAse E by blocking the sRNA-mRNA interaction remains unknown, but this could be a mechanism for avoiding degradation at low concentrations of sRNA [23]. Alternatively, the predicted ribosomal pauses at these sites might increase the chances of interactions between the sRNA and mRNA, inhibiting elongation of translation either by hitting an on/off switch or by enhancing mRNA degradation [24]. Whether pauses in translation are required for sRNAs to exert their regulatory actions within the mRNA coding region might be approached performing ribosome profiling experiments under the same culture conditions as Melamed et al. [9] analysis of sRNA-mRNA interactions were carried out.

## Discussion

sRNAs are small, noncoding RNAs that modify translation by preventing or enhancing the interaction between the ribosome and its binding site, or by modifying the translation efficiency of the target mRNA [25–27]. sRNA-mediated translational repression results in rapid degradation of the target mRNA, through Hfq-dependent recruitment of RNAse E. While the translational control mechanisms of sRNAs that target the 5’UTR of mRNAs are well understood, less is known about sRNAs that target the coding regions of mRNAs. Experimental characterization of Hfq-bound mRNAs and sRNAs has shown that coding regions are important targets of sRNAs [9, 28]. sRNAs that target the coding regions of mRNAs can modify mRNA stability without altering translation efficiency, through a RNAse E-dependent mechanism [29], as well as modulating translation efficiency without promoting mRNA degradation [30, 31].

Translation efficiency also can be influenced by the frequency and disposition of synonymous codons across the coding region, determining the rate and rhythm of translation [22, 32–34]. As both types of regulation are exerted in the coding sequence, our hypothesis was that these two mechanisms for altering elongation of translation might be related and complementary. To address this hypothesis, we used the information available in BSRD [13], which collects the targets of sRNA predicted by the IntaRNA [16] and RNAplex [35] computational algorithms, as well as the experimentally-validated sRNAs targets in sRNATarBase [15]. To our knowledge, this is the most complete database for *E. coli.* However, to carry out a broad analysis of sRNA-mRNA interactions in *E. coli*, it was necessary to complete the uncharacterized interactions, which represented 75% of the sRNA-mRNA pairs in BSRD. We used a bioinformatics approach to identify the sequences within the mRNAs that were targeted by sRNAs. We also used for a comparative analysis, the empirical data reported by Melamed et al. [9] on the Hfq-mediated interacting regions between sRNA and mRNAs. Our results indicated that most predicted interactions occurred near the middle of the mRNA coding regions, similar to findings obtained using empirical methods in other studies (9), reinforcing our initial hypothesis. However, comparisons between the codon preferences (as measured by CAI) of sRNA-regulated and non-sRNA-regulated mRNAs showed no significant differences between the two groups, even when this analysis was constrained to the mRNAs with interaction regions located in the middle of their coding sequences. When we split the coding sequence into separate subzones, we found that codon preference and adaptation to the tRNA pool were different in each zone, increasing from the coding DNA sequence near the 5’ end towards the central portion of the coding sequence. These results are consistent with the translational ramp previously described by Tuller et al. [22]. Our results showed that interaction regions tended to be rich in codons with limited tRNA available to decode them, which could suggest that sRNAs bind to the regions where the ribosomes tend to pause. These results could be biased by the interactions of sRNAs in the five codon window [36], which are also located in the translational ramp, rich in scarce codons with limited tRNA availability. sRNA interactions at the first five codons (CDS-5’) allow for repression of translation in the absence of any identifiable interaction by complementarity at the Shine-Dalgarno sequence or start codon, the classical mechanism of sRNA-mediated translation inhibition [37]. However, most interactions were located in the central region of the coding sequence, where CAI and tAI values are higher than in the CDS-5’ region. Further experiments are required to establish whether sRNA-mRNA interactions in coding regions require a slowing of ribosome translation. Whether the coding sequence constrains the sRNA-mRNA interaction is unknown. If this constraint exists, it would have a higher impact when sRNAs interact with multiple mRNAs.

Previous studies on sRNAs that recognize multiple targets have shown that interactions occur in different regions of the sRNA and that structural flexibility of the sRNA is required for interactions with different targets [38, 39]. To analyze multiple interactions, we built density maps of the interactions. These maps allowed for identification of sRNA regions that preferentially interact with groups of mRNAs. The profiles showed that most sRNAs have a modular structure, with some regions that interact preferentially with mRNAs. Although these characteristics have been previously described for Spot42 and FnrS [40, 41], our analyses showed that this feature is a general property of sRNAs that interact with multiple mRNAs. Our results also demonstrate the presence of conserved motifs in target mRNAs. Although previous studies have also identified conserved motifs in target mRNAs, our analysis shows that most of these motifs are present in coding regions, suggesting that the coding sequence imposes a constraining factor.

## Conclusions

Based on our analysis of the information available in BSRD, complemented with our own predictions of sRNA-mRNA interactions occurring within the mRNA coding sequences and the empirical data reported by Melamed et al. [9], we have shown that sRNAs preferentially recognize regions where tRNA availability is limited. As the binding of sRNAs to mRNA coding regions implies competition with the translating ribosome, this preference may have a dual effect. A reduced translation rate in these regions might induce pauses in the advancement or progression of the translating ribosome, preventing the sRNA-mRNA interaction and therefore RNAse E recruitment and mRNA degradation. Alternatively, such pauses might facilitate the sRNA-mRNA interaction, enhancing recruitment of RNase E and negatively regulating translation. These two alternative mechanisms may be strongly influenced by the availability of the aa-tRNAs that translate the interaction regions.

## Additional files

Additional file 1: Figures in Powerpoint format (ppt) showing density maps of the interaction of each sRNA with more than four targets. (PPTX 153 kb)

Additional file 2: Table in Excel format containing the information about sRNA, target mRNA, sRNA subzone, site of sRNA-mRNA interaction, CAI values, tAI values, and **Δ**G of sRNA-mRNA interaction using the data from BRSD. (XLSX 111 kb)

Additional file 3: Table in Word format (doc) showing sRNA zones and number of interaction in coding regions by zone and subzones for each sRNA with more than four interactions. (DOCX 14 kb)

Additional file 4: Figures in Powerpoint format (ppt) showing density maps of the interaction for each sRNA with multiple interactions in coding region. (PPTX 107 kb)

Additional file 5: Table in Excel format showing the number of interactions for each sRNA zones and for each site of interaction in target mRNAs. (XLSX 45 kb)

Additional file 6: Figure in Powerpoint format (ppt) showing the average CAI (A) and tAI (B) values of sRNA-intercating regions on mRNA coding sequences obtained from BSRDatabase (IR), from the Hfq-mediated sRNA-interacting regions reported by Melamed et al. (IE_E), and CDS-5’, (CDS-M), CDS-3’, and complete (CDS) coding sequences (as in Fig. 1). Comparison of CAI and tAI values were performed using non-parametric tests against IR (*) and IR_E (*). ***^/^*** *p* < 0.001. (PPTX 168 kb)

Additional file 7: Table in Excel format containing the information about target mRNAs, CAI and tAI values of mRNA interacting regions using the data from Melamed et al. (2016) [21]. (XLSX 538 kb)

## Abbreviations

BSRD: Small Regulatory RNA Database
CAI: Codon Adaptation Index
CDS: Coding sequences
IR: Interaction regions
mRNA: Messenger RNA
sRNA: Small RNA
tAI: tRNA Adaptation Index
tRNA: Transfer RNA
UTR: Untranslated region
