# Supplementary material for: Codon usage and modular interactions between messenger RNA coding regions and small RNAs in Escherichia coli
Source: BMC Genomics. 2018 Sep 6;19:657. doi: 10.1186/s12864-018-5038-6 (PMC6127932; doi:10.1186/s12864-018-5038-6)
Supplement: Supplementary file 1 — Figures in Powerpoint format (ppt) showing density maps of the interaction of each sRNA with more than four targets. (PPTX 153 kb) [file 12864_2018_5038_MOESM1_ESM.pptx]

## Slide 1
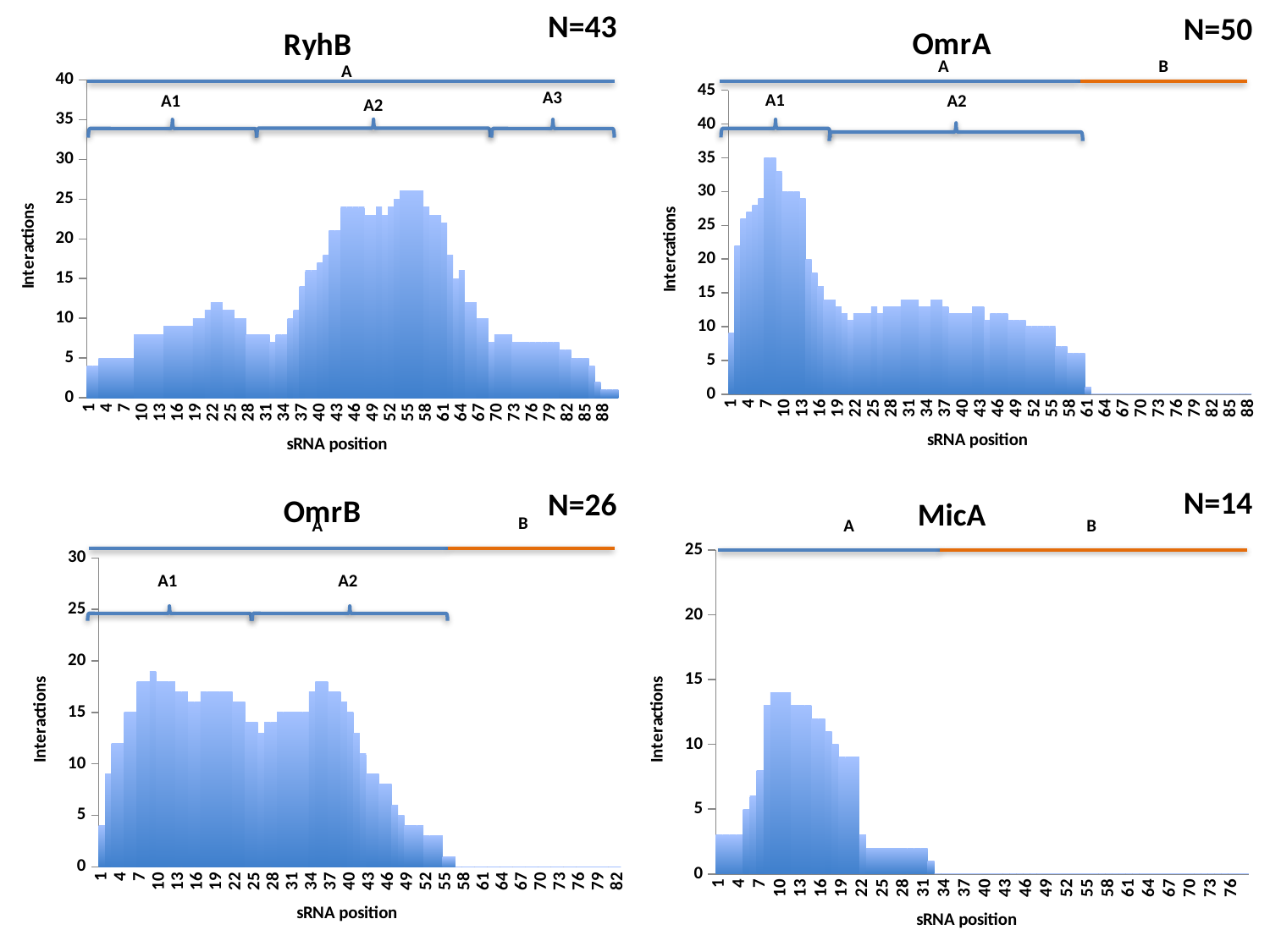

N=43
N=50
### Chart: OmrA
| Category | |
|---|---|
### Chart:
| Category | RyhB |
|---|---|A
A2
### Chart: OmrB
| Category | |
|---|---|
### Chart:
| Category | MicA |
|---|---|N=14
N=26

## Slide 2
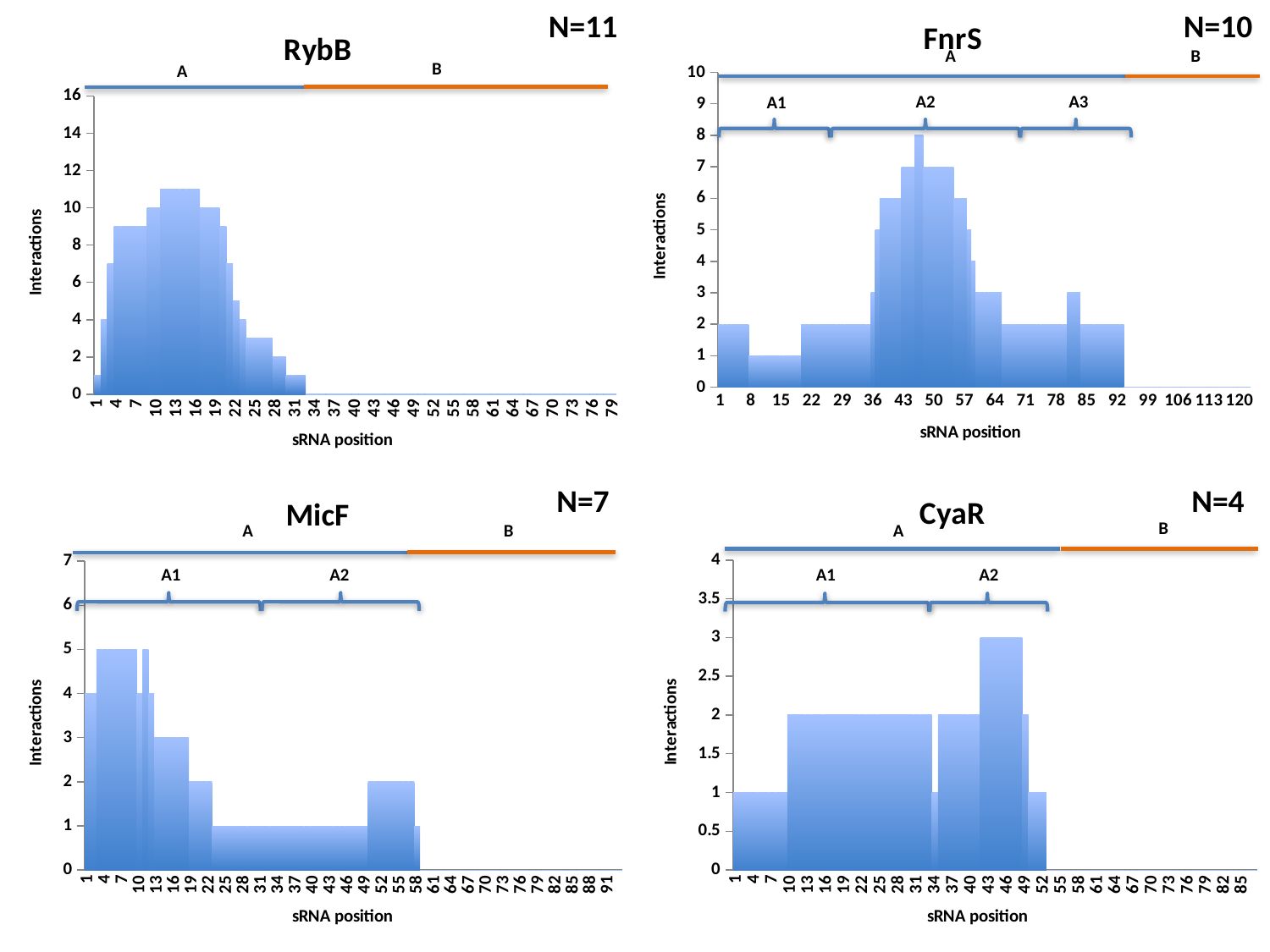

N=11
### Chart:
| Category | FnrS |
|---|---|N=10
### Chart:
| Category | RybB |
|---|---|B
A
N=7
### Chart:
| Category | CyaR |
|---|---|N=4
### Chart:
| Category | MicF |
|---|---|

## Slide 3
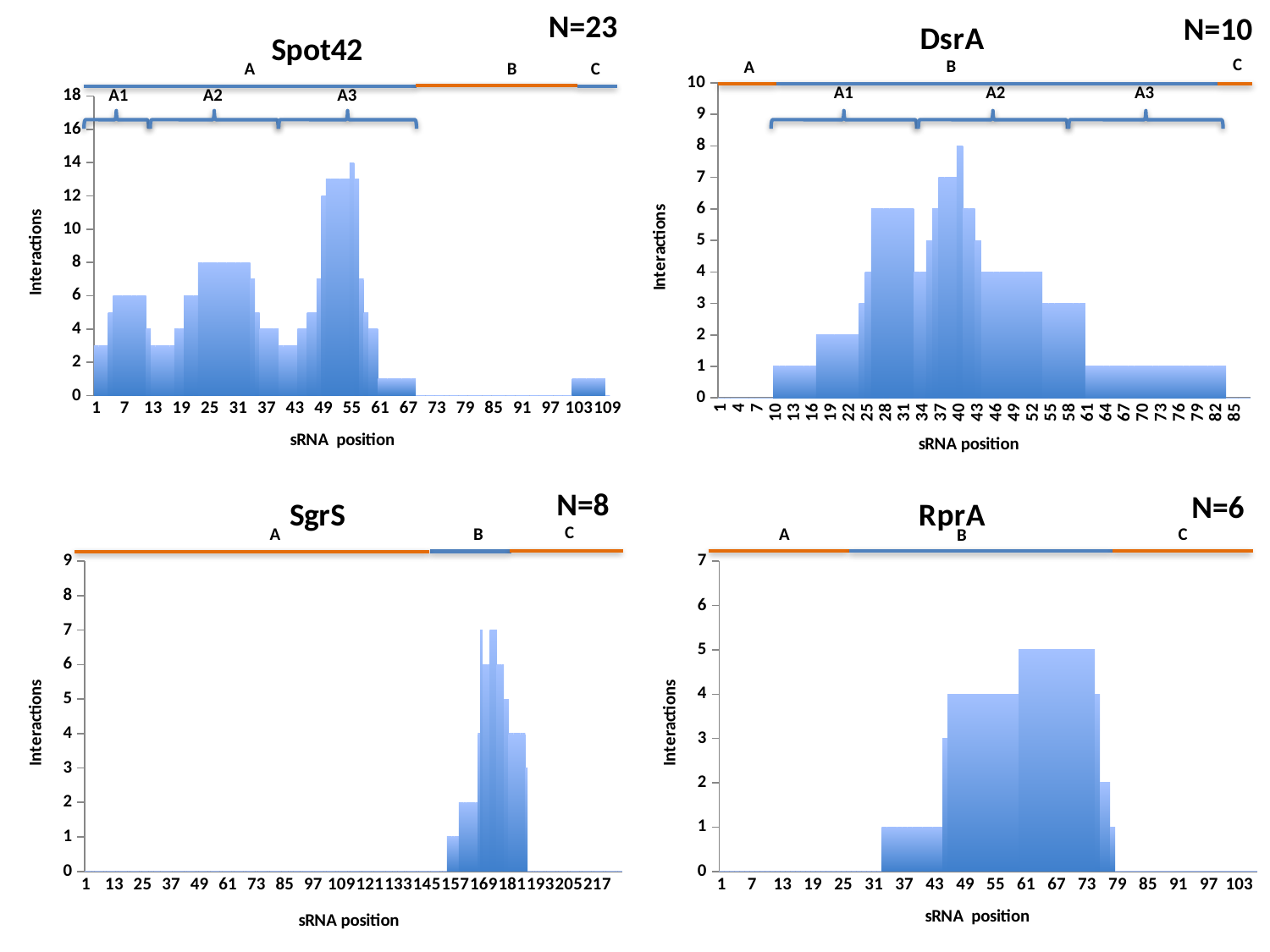

N=23
### Chart:
| Category | DsrA |
|---|---|N=10
### Chart: Spot42
| Category | |
|---|---|
### Chart:
| Category | SgrS |
|---|---|
### Chart:
| Category | RprA |
|---|---|N=8
N=6

## Slide 4
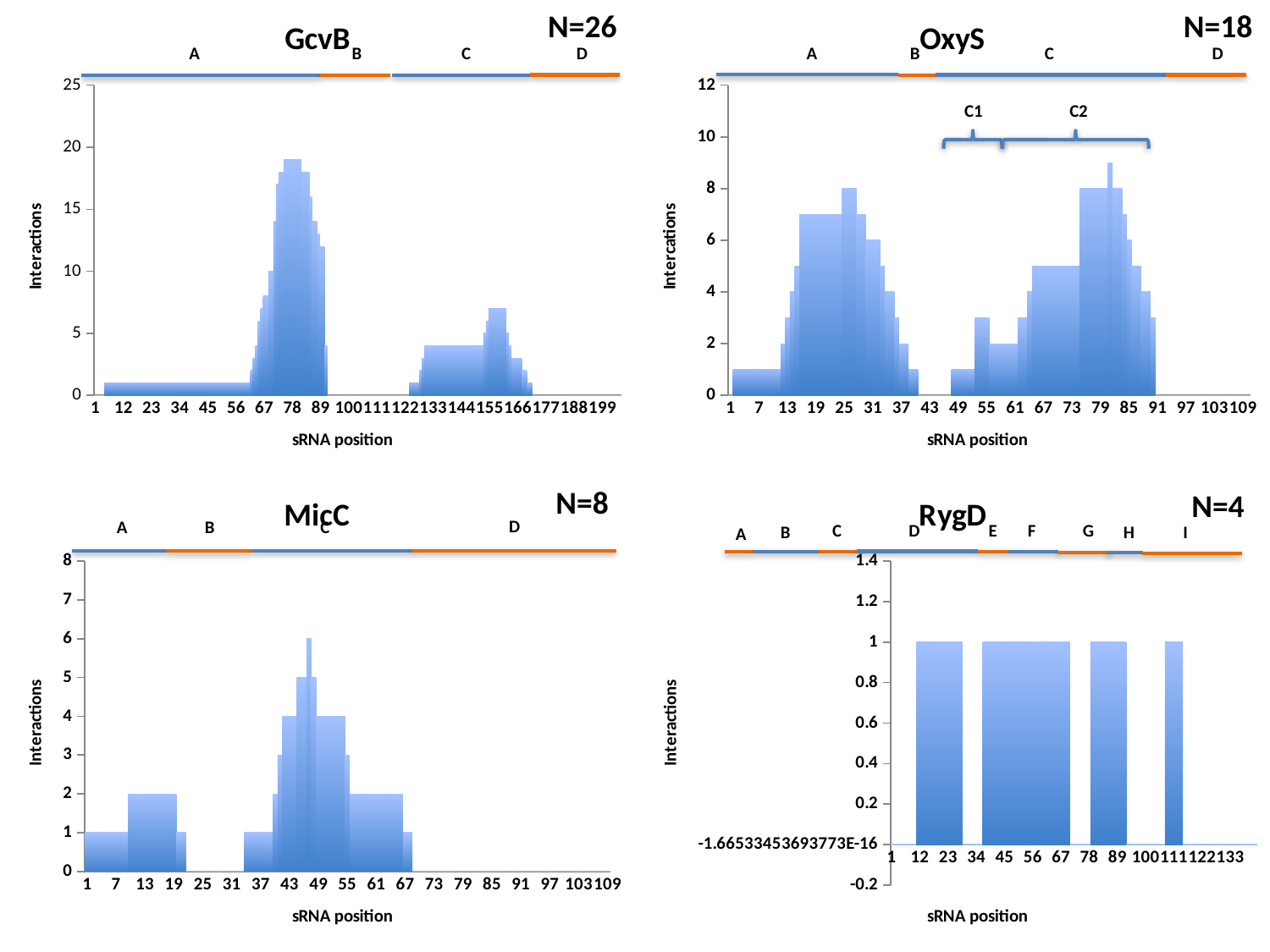

### Chart:
| Category | GcvB |
|---|---|N=26
### Chart: OxyS
| Category | |
|---|---|N=18
N=8
### Chart:
| Category | RygD |
|---|---|
### Chart:
| Category | MicC |
|---|---|N=4
