# Supplementary material for: Codon usage and modular interactions between messenger RNA coding regions and small RNAs in Escherichia coli
Source: BMC Genomics. 2018 Sep 6;19:657. doi: 10.1186/s12864-018-5038-6 (PMC6127932; doi:10.1186/s12864-018-5038-6)
Supplement: Supplementary file 3 — Table in Word format (doc) showing sRNA zones and number of interaction in coding regions by zone and subzones for each sRNA with more than four interactions. (DOCX 14 kb) [file 12864_2018_5038_MOESM3_ESM.docx]

| sRNA | Total Number Of targets | Zones (Number of Interactions) |
| --- | --- | --- |
| OmrA | 43 | A(35), B(17), C(0) |
| RyhB | 36 | A1(13), A2(27), A3(8) |
| OmrB | 18 | A1(14), A2(10), A3(12), B(0) |
| GcvB | 18 | A(0), B(14), C(0), D(4), E(0) |
| Spot42 | 17 | A1(3), A2(5), A3(13), B(0), C(1) |
| OxyS | 12 | A(0), B(5), C(0), D(7), E(0) |
| MicA | 10 | A(10), B(0) |
| RybB | 10 | A(10), B(0) |
| FnrS | 8 | A(0), B1(5), B2(3), C(0) |
| DsrA | 9 | A(0), B1(4), B2(8), B3(1), C(0) |
| SgrS | 7 | A(0), B(7), C(0) |
| MicC | 4 | A(0), B(1), C(0), D(3), E(0) |
| MicF | 5 | A(3), B(0), C(2), D(0) |
| RprA | 5 | A(0), B(5), C(0) |
| CyaR | 3 | A(0), B1(2), B2(2), C(0) |
| RygD | 3 | A(0), B(1), C(0), D(1), E(0), F(1), G(0) |
