# Supplementary material for: Codon usage and modular interactions between messenger RNA coding regions and small RNAs in Escherichia coli
Source: BMC Genomics. 2018 Sep 6;19:657. doi: 10.1186/s12864-018-5038-6 (PMC6127932; doi:10.1186/s12864-018-5038-6)
Supplement: Supplementary file 4 — Figures in Powerpoint format (ppt) showing density maps of the interaction for each sRNA with multiple interactions in coding region. (PPTX 107 kb) [file 12864_2018_5038_MOESM4_ESM.pptx]

## Slide 1
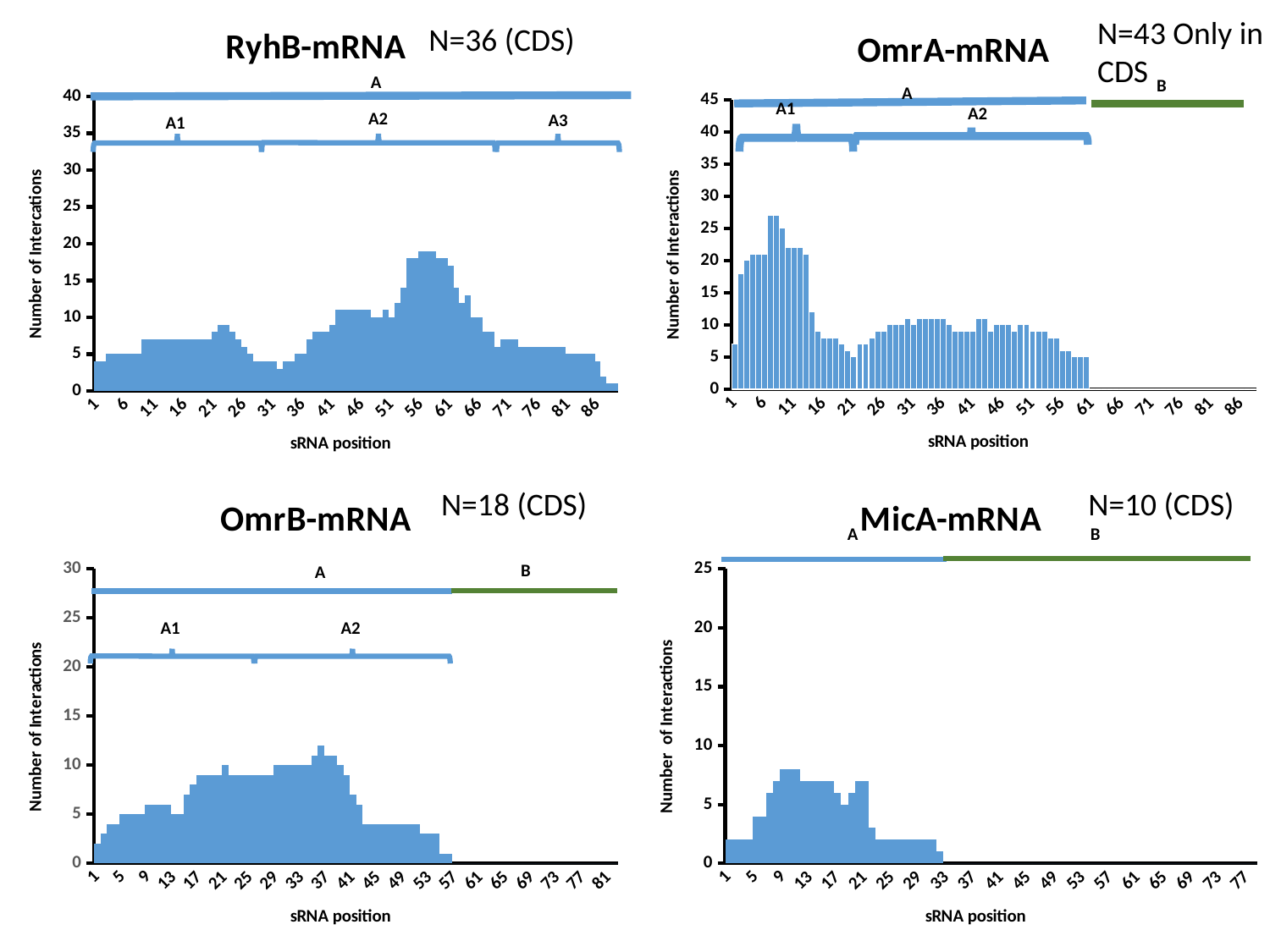

### Chart: RyhB-mRNA
| Category | |
|---|---|N=43 Only in CDS
### Chart: OmrA-mRNA
| Category | |
|---|---|N=36 (CDS)
A
B
A
A1
A2
A2
A3
A1
### Chart: OmrB-mRNA
| Category | |
|---|---|N=18 (CDS)
### Chart: MicA-mRNA
| Category | |
|---|---|N=10 (CDS)
B
A
A1
A2

## Slide 2
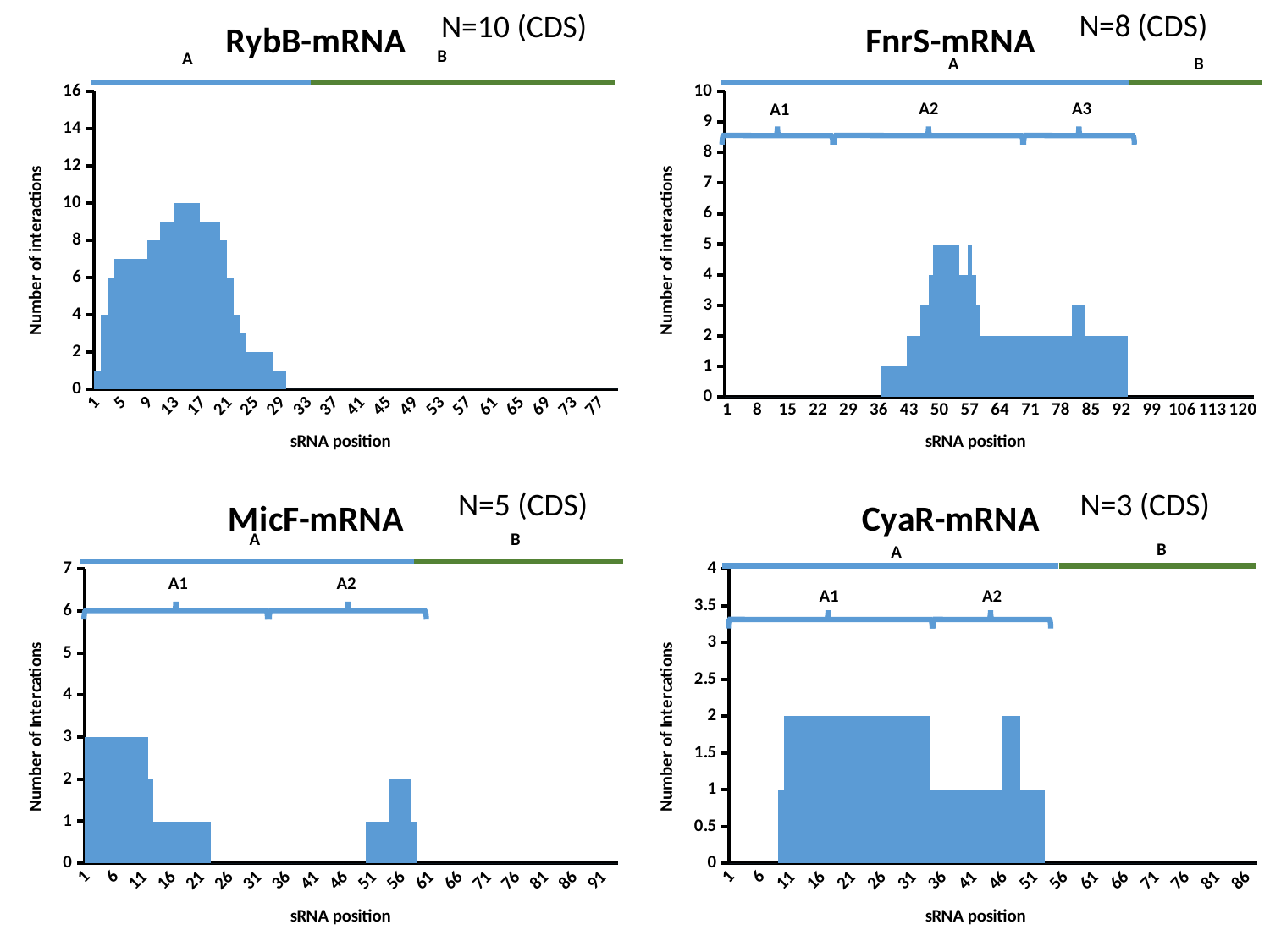

### Chart: RybB-mRNA
| Category | |
|---|---|N=10 (CDS)
### Chart: FnrS-mRNA
| Category | |
|---|---|
### Chart: MicF-mRNA
| Category | |
|---|---|N=5 (CDS)
### Chart: CyaR-mRNA
| Category | |
|---|---|N=3 (CDS)
B
A
A1
A2

## Slide 3
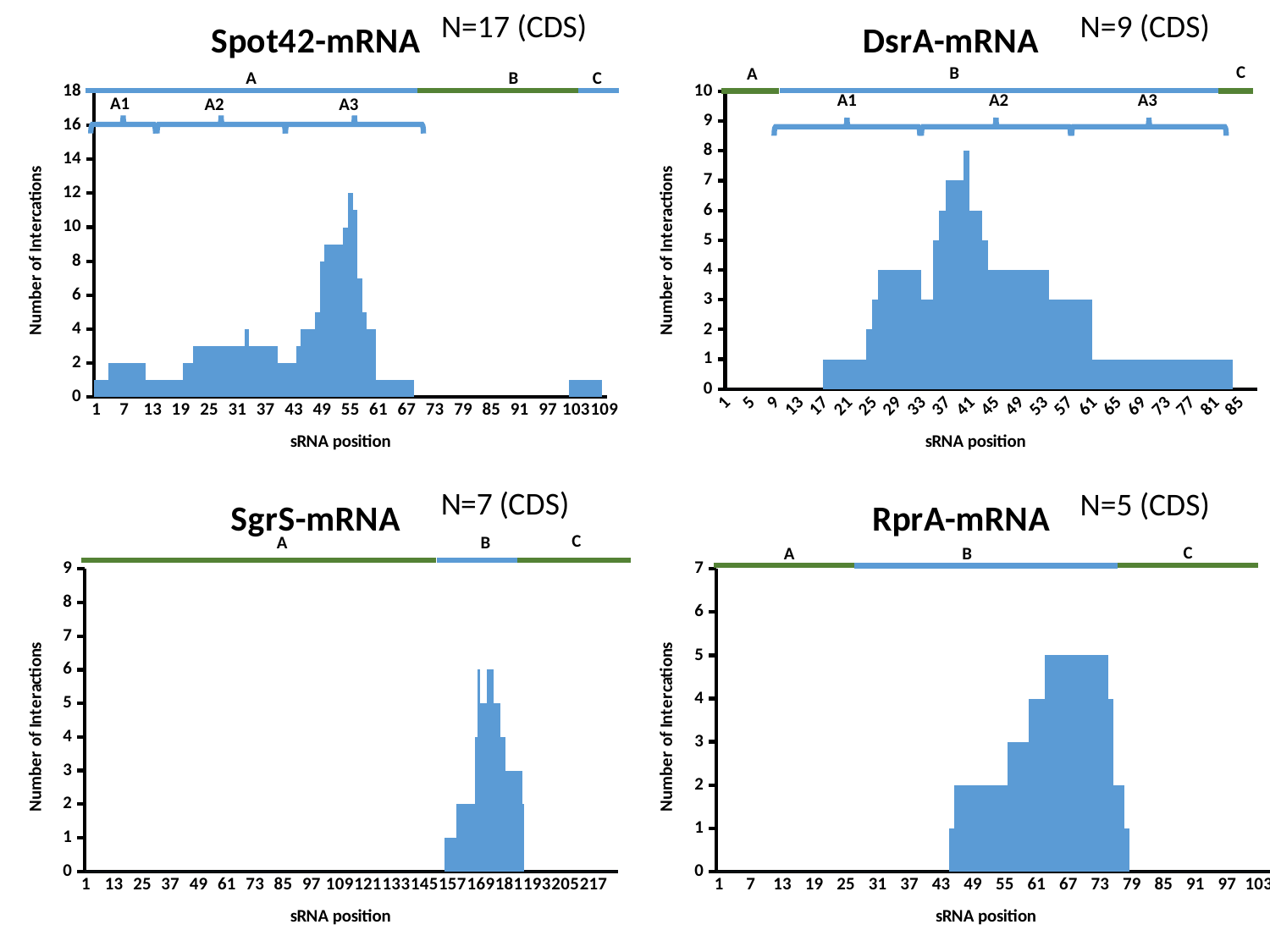

N=17 (CDS)
### Chart: DsrA-mRNA
| Category | |
|---|---|N=9 (CDS)
### Chart: Spot42-mRNA
| Category | |
|---|---|
A
B
C
A1
A2
A3
### Chart: SgrS-mRNA
| Category | |
|---|---|
### Chart: RprA-mRNA
| Category | |
|---|---|N=5 (CDS)
C
A
B

## Slide 4
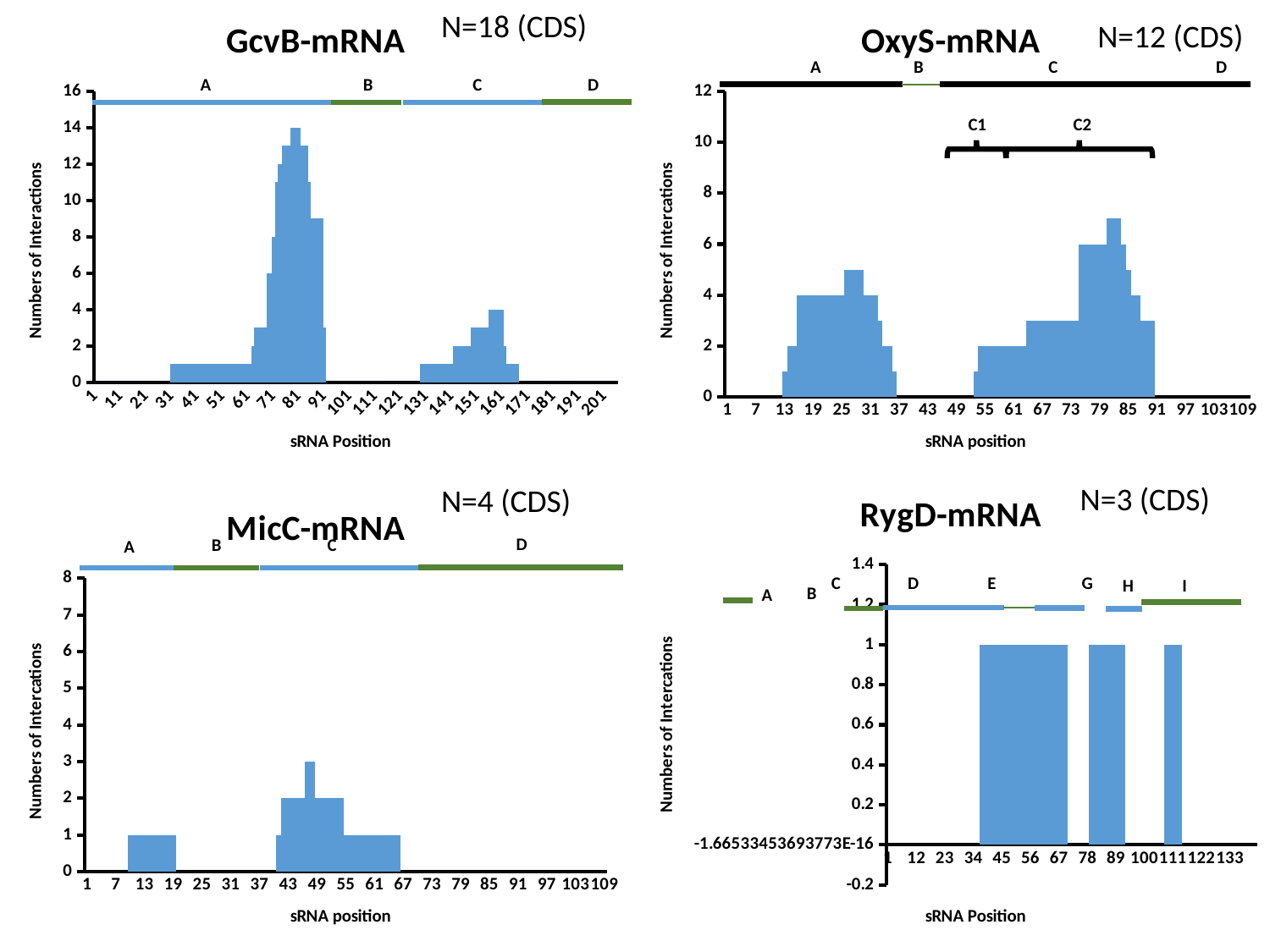

### Chart: GcvB-mRNA
| Category | |
|---|---|N=18 (CDS)
### Chart: OxyS-mRNA
| Category | |
|---|---|N=12 (CDS)
A
B
C
D
A
B
C
D
C1
C2
### Chart: RygD-mRNA
| Category | |
|---|---|N=3 (CDS)
N=4 (CDS)
### Chart: MicC-mRNA
| Category | |
|---|---|C
D
E
G
H
I
B
A
