# Supplementary figures and images for: Codon usage and modular interactions between messenger RNA coding regions and small RNAs in Escherichia coli
Source: BMC Genomics. 2018 Sep 6;19:657. doi: 10.1186/s12864-018-5038-6 (PMC6127932; doi:10.1186/s12864-018-5038-6)

## Slide 1
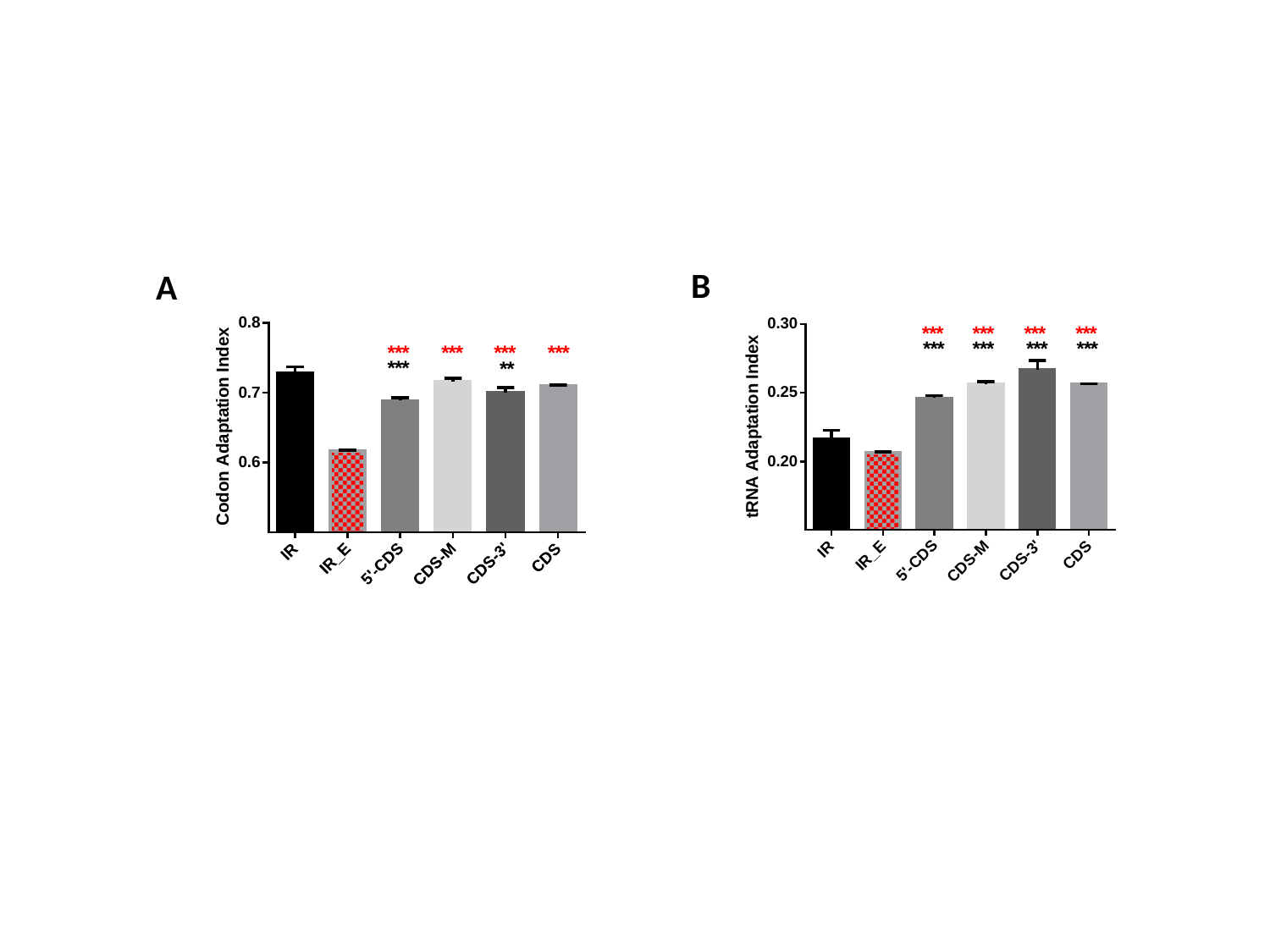

B
A

Supplement: Supplementary file 6 — Figure in Powerpoint format (ppt) showing the average CAI (A) and tAI (B) values of sRNA-intercating regions on mRNA coding sequences obtained from BSRDatabase (IR), from the Hfq-mediated sRNA-interacting regions reported by Melamed et al. (IE_E), and CDS-5’, (CDS-M), CDS-3’, and complete (CDS) coding sequences (as in Fig. 1). Comparison of CAI and tAI values were performed using non-parametric tests against IR (*) and IR_E (*). ***/*** p < 0.001. (PPTX 168 kb) [file 12864_2018_5038_MOESM6_ESM.pptx]
